# Implementing the semi-structured interview Kiddie-SADS-PL into an in-patient adolescent clinical setting: impact on frequency of diagnoses

**DOI:** 10.1186/1753-2000-2-14

**Published:** 2008-07-03

**Authors:** Bertrand Lauth, Sigurður Rafn A Levy, Guðlaug Júlíusdóttir, Pierre Ferrari, Hannes Pétursson

**Affiliations:** 1 University of Iceland, Landspítali University Hospital, Department of Child and Adolescent Psychiatry, Dalbraut 12, 105 Reykjavík, Iceland; 2 Université Pierre et Marie Curie, Ecole Doctorale 3C, 9 quai St Bernard, 75005 Paris, France; 3 University of Iceland, Landspitali University Hospital, Division of Psychiatry, Hringbraut, 101 Reykjavik, Iceland

## Abstract

**Background:**

Research is needed to establish the utility of diagnostic interviews in clinical settings. Studies comparing clinical diagnoses with diagnoses generated with structured instruments show generally low or moderate agreement and clinical diagnostic assignment (e.g. admission or chart diagnoses) are often considered to underdiagnose disorders. The objective of this study was to evaluate the impact of implementing the Schedule for Affective Disorders and Schizophrenia for School-Age Children – Present and Lifetime Version (Kiddie-SADS-PL) into an in-patient adolescent clinical setting.

**Methods:**

Participants were all adolescents admitted through the years 2001–2004 (*N *= 333 admissions, age 12–17 years). The authors reviewed the charts of the previous three years of consecutive admissions, patients being evaluated using routine psychiatric evaluation, before the Kiddie-SADS-PL was introduced. They then reviewed the charts of all consecutive admissions during the next twelve months, patients being evaluated by adding the instrument to routine practice.

**Results:**

The rates of several main diagnostic categories (depressive, anxiety, bipolar and disruptive disorders) increased considerably, suggesting that those disorders were likely underreported when using non-structured routine assessment procedures. The rate of co-morbidity increased markedly as the number of diagnoses assigned to each patient increased.

**Conclusion:**

The major differences in diagnostic assignment rates provide arguments for the utility of diagnostic interviews in inpatient clinical settings but need further research, especially on factors that affect clinical diagnostic assignment in "real world" settings.

## Background

Formal DSM [1] or ICD [2] diagnoses are now required for admission and treatment in most mental health facilities and settings. In addition the diagnoses generated for this purpose also figure prominently in treatment planning, as clinicians shape interventions to address the diagnoses assigned [3].

But research does not support unstructured interviews as reliable means to standard diagnoses. When colleagues of the same discipline working in the same clinical setting were often unable to agree about an individual's diagnosis even when they were presented with exactly the same information, researchers concluded that this situation needed to be remediated and began to develop *structured interviews *[3,4].

Even experienced clinical interviewers are not reliable diagnosticians when compared which each other or when compared with structured interviews.

Tables 1 and 2 present a review of studies on agreement between clinicians' diagnoses and diagnoses generated with standardized interview procedures in child and adolescent psychiatry. These studies use generally J Cohen's *kappa *[5] and concern both inpatient and outpatient as well as community populations.

**Table 1:** Studies comparing clinical diagnoses with diagnoses generated with diagnostic interviews

**STUDY**	**INSTRUMENT**	**Sample Size**	**Dx**	**Median kappa**	**Range**
***Inpatients***					
Carlson et al.(1987) [28]	K-SADS-P	30	6	.50	.16 to .69
	DICA (Child version)			.38	.15 to .75
	DICA-P (Parent version)			.40	.05 to .66
Welner et al.(1987) [29]	DICA-C (Child version)	27	5*	.26	-.18 to .52
Apter et al.(1989) [30]	K-SADS-P (Hebrew)	70	6*	.64	NA
Weinstein et al.(1989) [31]	DISC-1	163	6*	.09	.03 to .17
Aronen et al.(1993) [32]	"	"	6*	.09	-.07 to .22
Vitiello et al.(1990) [33]	DICA-C	46	3*	.28	-.03 to .62
	DICA-P			.28	.10 to .48
Shanee et al.(1997) [34]	K-SADS-PL (Hebrew)	57	19	.80	.48 to 1.00
Fristad et al.(1998) [35]	CHIPS	47	15	.51	.31 to .78
Pellegrino et al.(1999) [36]	DISC-R 2.1	50	5	.09	.03 to .61

Note: Dx = Number of disorders on which the standard kappas were calculated

*Broad diagnostic clusters

• K-SADS-P = Schedule for Affective Disorders and Schizophrenia for School-Age Children-Present Episode Version (K-SADS-P) [9]. K-SADS-PL = Present and Lifetime version [37].

• DICA = Diagnostic Interview Schedule for Children and Adolescents [38]. DICA-C = child version. DICA-P = Parent version [39,40]. DICA-R = Revised version [41-43].

• CHIPS = Children's Interview for Psychiatric Syndromes [44].

• DISC-1 = Diagnostic Interview Schedule for Children [45]. DISC-R 2.1 = second revision [46]. DISC 2.3 = version 2.3 [47]. DISC-IV = version IV [48].

**Table 2:** Studies comparing clinical diagnoses with diagnoses generated with diagnostic interviews (cont.)

**STUDY**	**INSTRUMENT**	**Sample Size**	**Dx**	**Median kappa**	**Range**
***Community sample***					
Bird et al.(1992) [49]	DISC-C (Spanish)	386	5	.29	.04 to .42
	DISC-P (")			.39	.24 to .50
Schwab-Stone (1996) [50]	DISC 2.3 – Parent	247	8	.47	.29 to .74
	" – Youth			.33	.27 to .77
	" – Combined			.49	.40 to .80

***Outpatients***					
Rubio-Stipec et al.(1994) [51]	DISC-2 (Spanish) – Parent	322	7*	.32	.07 to .58
	" – Youth			.29	-.02 to .54
Ezpeleta et al.(1997) [52]	DICA-R (Spanish) – Child	137	14	.31	-.04 to 1.00
	" – Adolescent			.31	.07 to .55
	" – Parent			.41	-.02 to 1.00
Fristad et al.(1998) [53]	CHIPS – Parent version	21**	14	.49	.03 to .81
Teare et al.(1998) [54]	" – Youth version	26**	12	.45	.01 to .72
Jensen et al.(2002) [3]	DISC-P 2.3	245	10	.06	-.03 to .27
			5*	.09	.00 to .44
Lewczyk et al.(2003) [55]	DISC-IV	240	4*	.09	-.04 to .22
Kim et al.(2004) [56]	K-SADS-PL (Korean)	91	5*	.41	.24 to .69
Ghanizadeh et al.(2006) [57]	K-SADS-PL (Farsi)	109	16	.82	.49 to 1.00

Note: Dx = Number of disorders on which the standard kappas were calculated

*Broad diagnostic clusters; **In- and outpatients

Our review consistently revealed low or moderate levels of agreement, except for K-SADS. Additionally, findings showing poor agreement were generally robust across multiple methodological variations, as for instance assessing agreement at the level of broader diagnostic clusters. Agreement is usually higher for externalizing diagnostic categories than for internalizing ones and many authors suggest that diagnoses of anxiety and depression can be missed using an unstructured interview.

Research is still needed to establish the utility of diagnostic interviews in clinical settings. By encouraging clinicians to follow standard diagnostic and interviewing methods, structured interviews promote more consistent diagnostic practices and help justify therapeutic interventions and outcomes. Miller [6,7] suggested that because the structured interview yields precise diagnostic data, appropriate treatments may be delivered earlier, leading to more rapid recovery and shorter hospital stays.

However, the instrument is not a substitute for clinical judgement. As McClellan and Werry [8] pointed out, psychiatric decision making depends on the integration of informations from diverse sources and perspectives, including the patient and family interviews, the mental status examination, collateral informants (teachers) and other treatment providers. The pre-eminent role of the clinician must be recognized and preserved.

In in-patient clinical settings, it is of particular importance that diagnoses can be reliably made. Adolescents who need admission in a psychiatric unit are often seriously disturbed and show considerable impairment. Many of them need acute admission because the assessing clinician has detected a significant suicidal risk. In this context, the project of implementing a semi-structured diagnostic interview (here the K-SADS-PL) into clinical practice has been welcomed by the staff members as well as by parents. The majority expected the instrument to provide more precise nosological data, so that diagnoses could be more reliable and appropriate treatment would be delivered earlier.

### Aim

To evaluate the impact that introducing the interview had on diagnoses, we reviewed charts of the previous three years of consecutively admitted patients evaluated using routine psychiatric evaluation, before we started using the Kiddie-SADS-PL. Then we reviewed the charts of the next twelve months, patients being evaluated by adding the instrument to routine practice.

## Methods

### Clinical context

The adolescent unit of the Department of Child and Adolescent Psychiatry of the Landspítali University Hospital in Reykjavík, is the only psychiatric ward for adolescents in Iceland, admitting each year between 70 and 80 patients from 12 to 17 years of age, from all parts of the country. The main reasons for admission are severe behavioural and/or emotional disturbances with severe functional impairment and often suicidality (61% of cases in the period 2001–2004), 53% being acute admissions. Mean length of stay is 43 days for the period 2001–2004 (SD = 46.74). Adolescents presenting with alcohol and drug abuse as a predominant problem are referred to other service providers, such as social and child welfare services. The population admitted is culturally homogeneous and its geographic distribution throughout the eight regions of the country is representative of the general population for the same age category (12 to 17 years old). Since the unit is the only facility in the country providing psychiatric in-patient treatment for adolescents, we assume that our population is representative of the most severe range of psychiatric morbidity of the adolescent clinical population in Iceland.

### Participants

Participants were all adolescents admitted to the in-patient unit of the Department of Child and Adolescent Psychiatry, Landspítali University Hospital in Reykjavík, through the years 2001, 2002, 2003 and 2004 (*N *= 333 admissions). Boys: 43% (*n *= 144); Girls: 57% (*n *= 189). Age: 12 to 17 years-old (Mean: 14.8; SD = 1.33), 71% are between 14 and 16.

The study was approved by the Data Protection Authority and the National Bioethics Comittee in Iceland.

### Measures

Clinical diagnoses were made on the basis of admission history, mental status, nursing obervations, psychometric and psychoeducational testing and treatment course. Consensus diagnoses were used and in case of disagreement, discussion with ward staff members helped to clarify information in the chart. Diagnoses were assigned according to the symptomatology present at the time of admission.

The Kiddie-SADS interview has changed since its original publication [9] and is currently available in different *DSM-IV *format versions: the Kiddie-SADS-P IVR (*Present State*), the Kiddie-SADS-L (Lifetime), the Kiddie-SADS-PL (*Present and Lifetime Version*) and the Kiddie-SADS-E (*Epidemiological*) [10]. The present study used the K-SADS-PL, which has several strengths [11]. It has strong content validity because it was designed to tap pre-specified diagnostic criteria and includes detailed probes useful in eliciting clinically meaningful information. It is also the only instrument that provides global and diagnosis-specific impairment ratings to facilitate the determination of "caseness". In addition, the Kiddie-SADS-PL provides a clinician-friendly front and screening examination which may result in a more efficient shorter interview.

The Icelandic version of the K-SADS-PL was developed by classic translation-back translation technique with a bilingual expert committee assessing equivalence in several dimensions [12].

The inter-rater reliability of the Icelandic version was assessed by re-rating 15 randomly selected interviews [12]. Experienced and trained clinicians rated the interviews independently either by videotape or by attending the interview session (three clinical psychologists and one child and adolescent psychiatrist took part in the project). Reliability was satisfactory for most diagnostic categories (kappa = .44 – 1.00), except for Mania (.31). We also examined inter-rater reliability at the symptom level separately for each diagnostic area, with kappa values calculated for each item. Average values at the symptom level within each diagnostic category ranged from .48 (Social Phobia) to .98 (Oppositional Defiant Disorder).

Additionally, we obtained correlations of *numbers of diagnostic criteria met *between raters and found them satisfactory in all diagnostic categories (Pearson's *r *= .76 – 1.00).

Finally, we examined inter-rater reliability across main diagnostic areas surveyed in the screen interview, in order to estimate agreement in utilization of skip-out criteria. The average agreement evaluated by calculation of Kappa statistics across the diagnostic areas studied was .90 (range = .57 through 1.00).

The convergent and divergent validity of the skip-out screens and most frequent diagnoses generated with the Icelandic version of the K-SADS-PL was determined in another study using an adolescent clinical inpatient sample (*N *= 86) against eleven standard self-report or parent-report rating scales which had already been translated, adapted and in most cases validated in Iceland: rating scales of depression [13,14], anxiety [15,16], ADHD [17,18], behavioral and other psychiatric problems [17,19-21]. The results indicated that the Icelandic version of K-SADS-PL generates valid *DSM-IV *depression, anxiety and behavioral diagnoses in severely affected adolescent in-patients. Divergent validity was only partially supported in our very comorbid clinical sample.

### Procedure

1. We reviewed clinical charts of the previous three years of consecutively admitted patients evaluated using routine non-structured psychiatric evaluation (*N *= 248). Assessments had been made with unstructured clinical interviews and consensus observation within the unit, the *ICD-10 *diagnoses being assigned by six experienced child and adolescent psychiatrists and appearing in the records, as well as assessment of suicidality. Diagnoses had been assigned according to the symptomatology present at the time of admission.

2. Then we reviewed charts of all consecutive admissions during the next twelve months (*N *= 85), patients being evaluated with the structured interview Kiddie-SADS-PL in addition to routine diagnostic procedures. As the main official diagnostic classification system in European countries is *ICD-10 *for both clinical and research purposes, results of Kiddie-SADS interviews algorithms have been translated into *ICD-10*. A few additional questions were included in the interviews for *ICD-10 *criteria not covered by the K-SADS-PL [22].

Two coders checked to verify accurate utilization of *DSM-IV *and *ICD-10 *algorithms for assignment of final diagnosis. Suicidality was assessed with the diagnostic interview.

In both study periods, combined diagnoses according to *ICD-10 *(F41.2, F90.1 and F92) were categorized as two co-morbid disorders.

The comparability of the two populations was evaluated by reviewing variables other than diagnoses: age, sex, geographic distribution, mean length of admission and several risk factors:

- parents' separation or divorce

- having moved or changed school more than twice during the six months prior to admission

- a history of being bullied during the six months prior to admission

- a confirmed history of child neglect, physical, sexual or emotional abuse

- at least one parent having a confirmed history of psychiatric disease

- at least one parent having a confirmed history of mental and behavioural disorder due to psychoactive substance use

These risk factors were identified during admission with clinical interviews and detailed and comprehensive evaluation of the patient's history. Only documented and reported cases of child abuse or neglect were considered. Definitions of the various maltreatment categories are those commonly accepted [23].

Six patients could not be evaluated with the Kiddie-SADS (Four with Autism or related Pervasive Developmental Disorders, one with Mental Retardation and one with severe Language Disorder), and in four cases parents didn't participate or couldn't be reached for interviews. In twelve other cases, only parents could be interviewed and diagnoses were generated by combining information collected with routine patient evaluation in the unit. In all other cases, diagnoses were generated by combining information from parents' interview with information from adolescent interview and routine patient evaluation.

The first author of the study was the only Kiddie-SADS interviewer.

The adolescents' interviews (*n *= 63) were conducted on the day of admission in 5 cases (7.9%), but 73 days after admission in one case (interval of time: 0 to 73 days, mean = 13.4, *SD *= 13.3; median = 9).

The parents' interviews (*n *= 75) were conducted on the day of admission in 4 cases (5.3%), but 71 days after admission in one case (interval of time: 0 to 71 days, mean = 11.0, *SD *= 12.9; median = 6).

Diagnoses were assigned according to the symptomatology present at the time of admission.

### Statistical analysis

The Statistical Package for Social Sciences (SPSS) was used for data analysis. For comparisons between groups on categorical variables (sex, geographic area, presence of risk factors, assigned diagnoses), Chi-Square tests were applied and odds ratios were calculated. Independent-samples t-tests were conducted to compare groups on continuous variables (age, mean length of admission).

## Results

### Comparability of the two populations

There were no statistically significant differences (p > .05) between the two groups according to the following variables:

- Age and mean length of admission (Table 3)

**Table 3:** Comparability of the two populations: age and mean length of admission

***Variables***	**Mean**	**SD**	***t***	***p***
Age (2001–2003, n = 248)	14.78	1.36	.60	.55
Age (2004, n = 85)	14.88	1.25	.60	.55
Mean length of admission (2001–2003, n = 248)	40.83	46.04	1.38	.17
Mean length of admission (2004, n = 85)	48.95	48.52	1.38	.17

Note: Significance on a 5% level

- Sex and 9 risk factors listed before (Table 4)

**Table 4:** Comparability of the two populations: sex and risk factors

	**2001–2003 **	**2004 **			
***Variables***			**OR**	**CI**	***p***
	**(*N *= 248)**	**(*N *= 85)**
Sex (males/females)	107/141	37/48	.98	.60–1.62	1.00
Parents' separation or divorce	114	34	.78	.47–1.29	.41
Having moved or changed school more than twice*	19	7	1.08	.44–2.67	.87
Bullied at school*	87	31	1.06	.64–1.77	.92
Confirmed history of neglect	119	34	.72	.44–1.19	.25
Confirm. history of emotional abuse	63	16	.68	.37–1.26	.28
Confirm. history of physical abuse	25	10	1.19	.55–2.59	.82
Confirmed history of sexual abuse	71	15	.53	.29–.99	.06
One parent with diagnosed psychiatric disease	76	22	.79	.45–1.38	.49
One parent with diagnosed alcohol or substance abuse disorder	69	20	.80	.45–1.42	.53

Note: OR = Odds Ratio; CI = 95% Confidence Interval; Significance on a 5% level

*during last 6 months

- Geographic distribution: the odds ratios for the 8 different regions ranged from .29 to 2.38; the proportion of patients from Reykjavík vs. other (rural) regions slightly increased between the two study period (from 63.7% to 70.6%).

### Impact on diagnoses

We observed considerable changes in the frequencies of several diagnoses and in co-morbidity rates (Table 5).

**Table 5:** Comparison of prevalences in clinical samples without and with K-SADS

	**2001–2003 **	**2004 **			
***Diagnostic or symptom categories***			**OR**	**CI**	***p***
	**(N = 248)**	**(N = 85)**
Alcohol and/or drug abuse	36 (14.5%)	7 (8.2%)	.53	.23–1.24	.19
Psychotic or Bipolar disorders	24 (9.7%)	19 (22.4%)	2.69	1.39–5.21	.00
Depressive disorders	79 (31.9%)	49 (57.6%)	2.91	1.75–4.83	.00
Any anxiety disorder	74 (29.8%)	43 (50.6%)	2.41	1.45–3.99	.00
Separation anxiety disorder	4 (1.6%)	13 (15.3%)	11.01	3.48–34.82	.00
Social phobia/Social anxiety dis.	17 (6.9%)	17 (20.0%)	3.40	1.65–7.01	.00
Overanxious/Generalized anxiety disorder	23 (9.3%)	21 (24.7%)	3.21	1.67–6.17	.00
Stress and adjustment disorders	86 (34.7%)	29 (34.1%)	.97	.58–1.64	1.00
Eating disorders	10 (4.0%)	12 (14.1%)	3.91	1.62–9.42	.00
Any behavioural disorder	97 (39.1%)	45 (52.9%)	1.75	1.07–2.88	.04
Any Hyperkinetic disorder	52 (21.0%)	21 (24.7%)	1.24	.69–2.21	.57
Oppositional defiant and conduct disorders	63 (25.4%)	35 (41.2%)	2.06	1.22–3.45	.01
Suicide attempt or self-harm	94 (37.9%)	34 (40.0%)	1.09	.66–1.81	.83
Suicidal ideation	83 (33.5%)	45 (52.9%)	2.24	1.35–3.69	.00

Note: OR = Odds Ratio; CI = 95% Confidence Interval; Significance on a 5% level

The mean number of diagnoses assigned to each patient admitted rose from 2.4 (*SD *= 1.2) to 3.4 (*SD *= 1.5). Only 13% of all admissions received only one diagnosis during the second period of the study, against 29% during the first period.

We observed a very significant increase in the number of patients diagnosed with depressive disorders (*ICD-10 Severe depressive episode F32.2, Moderate depressive episode F32.1, Dysthymia F34.1*) and with anxiety disorders (*ICD-10 Phobic anxiety disorder of childhood F93.1, Social anxiety disorder of childhood/Social phobia F93.2/F40.1, Separation anxiety disorder of childhood F93.0, Overanxious/Generalized anxiety disorder of childhood F93.8/F41.1*).

The number of patients diagnosed with *Eating disorders F50 *increased significantly.

We observed also a significant increase in the rate of patients diagnosed with psychotic and bipolar disorders (*ICD-10 Bipolar affective disorder F31, Manic episode F30, Acute and transient psychotic disorders F23*) and with any behavioural disorder but this was only because of *Oppositional defiant disorder *and other conduct disorders F91; the rate of patients diagnosed with *Hyperkinetic disorders F90 *didn't increase significantly between the two study periods.

There was no significant change in the number of patients diagnosed with *Mental and behavioural disorders due to psychoactive substance use F10–F19*, and *Stress and adjustment disorders F43*.

The rate of suicide attempts or self-harm didn't change significantly between the two periods but the rate of patients detected with suicidal thoughts increased dramatically with the use of the semi-structured diagnostic interview.

## Discussion

This study was conducted to assess the impact of implementing a structured interview on diagnoses, in a clinical population of severely affected adolescents presenting a range of symptoms that suggest multiple diagnostic possibilities. The results show considerable increase in the rates of patients diagnosed with several main diagnostic categories (e.g. depressive, anxiety, bipolar and disruptive disorders) suggesting that those disorders were likely under-reported when using unstructured conventional routine assessment procedures. The rate of co-morbidity increased markedly. Using the structured diagnostic instrument, the non-specific diagnoses "*Other" *or "*Unspecified" *were much less frequently assigned and diagnoses were made that had probably been missed before. Importantly, the interview helped to detect suicidal ideation.

Under-evaluation of depressive disorders in a clinical population has been reported before [22], as well as under-detection of anxiety disorders [24] or bipolar disorders [25,26].

But we must be aware that there may have been changes of the patients' characteristics between the both time periods of our study. This bias is however limited by the fact that several variables other than diagnoses have been reviewed to evaluate the comparability of the two samples.

Additionally the first author of the study was the only Kiddie-SADS interviewer, which could constitute a bias. He was also one of the clinicians assigning diagnoses during the first period, which limits the validity of our comparisons. These biases are however limited by the fact that an evaluation of inter-rater reliability indicated a high rate of agreement between the interviewer and other experienced clinicians.

It has been our experience that adding the standardized diagnostic instrument allowed more precise diagnostic evaluations; some authors [6,7] have suggested that this may lead to more appropriate treatment delivery. Despite the time needed for interviews, patients and their families usually reacted positively, feeling satisfied that they were evaluated thouroughly, which was in line with other studies [27]. Traditional psychiatric evaluations and psychodynamic formulations were not abandonned, but the highly detailed symptomatic assessment helped the staff and therapists to set up cognitive and behavioural interventions during and after admissions.

In the present study, the introduction of the structured diagnostic interview has led the clinician assigning diagnoses to use more extensively the concept of co-morbidity, according to *DSM-IV *classification diagnostic system, and abandon the *ICD-10 *philosophy of emphasizing one main diagnosis in each patient.

## Conclusion

This study provides arguments for the utility of diagnostic interviews in inpatient clinical settings. The major differences in diagnostic assignment rates between the two periods are in line with other research findings that have suggested a mismatch between diagnoses in practice and diagnoses in research. A useful objective for future studies will be to understand this mismatch and how to address it. Major changes in diagnostic rates underline the need for better understanding of factors that affect clinical diagnostic assignment in "real world" settings.

## Competing interests

The authors declare that they have no competing interests.

## Authors' contributions

BL: conception, principal investigator, designer, statistical analysis, interpretation. SRL: conception, designer, principal investigator, statistical analysis, interpretation. GJ: investigator, interpretation. PF: conception, designer, statistical analysis, interpretation, revision. HP: conception, designer, statistical analysis, interpretation, revision. All authors read and approved the final manuscript.

## References

[B1] American Psychiatric Association (1994). Diagnostic and Statistical Manual of Mental Disorders Washington DC.

[B2] World Health Organisation (1992). The International Classification of Diseases Geneva.

[B3] Jensen AL, Weisz JR (2002). Assessing match and mismatch between practitioner-generated and standardized interview-generated diagnoses for clinic-referred children and adolescents. J Consult Clin Psychol.

[B4] Cantwell D, Rutter M, Tuma A, Lann L (1988). DSM-III studies. Assessment and diagnosis in child psychopathology.

[B5] Cohen J (1960). A Coefficient of Agreement for Nominal Scales. Educational and Psychological Measurement.

[B6] Miller PR (2001). Inpatient diagnostic assessments: 2. Interrater reliability and outcomes of structured vs. unstructured interviews. Psychiatry Research.

[B7] Miller PR, Dasher R, Collins R, Griffiths P, Brown F (2001). Inpatient diagnostic assessments: 1. Accuracy of structured vs. unstructured interviews. Psychiatry Research.

[B8] McClellan JM, Werry JS (2000). Introduction – research psychiatric diagnostic interviews for children and adolescents. J Am Acad Child Adolesc Psychiatry.

[B9] Chambers WJ, Puig-Antich J, Hirsch M, Paez P, Ambrosini PJ, Tabrizi MA, Davies M (1985). The assessment of affective disorders in children and adolescents by semistructured interview. Test-retest reliability of the schedule for affective disorders and schizophrenia for school-age children, present episode version. Arch Gen Psychiatry.

[B10] Ambrosini PJ (2000). Historical development and present status of the schedule for affective disorders and schizophrenia for school-age children (K-SADS). J Am Acad Child Adolesc Psychiatry.

[B11] Kaufman J, Schweder AE, Hersen M, Segal DM, Hilsenroth M (2003). The Schedule for Affective Disorders and Schizophrenia for School Age Children: Present and Lifetime Version (K-SADS-PL). The Comprehensive Handbook of Psychological Assessment (CHOPA), Volume 2: Personality Assessment.

[B12] Lauth B, Magnússon P, Ferrari P, Pétursson H An Icelandic version of the Kiddie-SADS-PL: Translation, cross-cultural adaptation and inter-rater reliability. Nord J Psychiatry.

[B13] Kovacs M (1992). Children's Depression Inventory (CDI) manual.

[B14] Beck JS, Beck AT, Jolly J (2001). Manual for the Beck Youth Inventories of Emotional and Social Impairment.

[B15] Beck AT, Epstein N, Brown G, Steer RA (1988). An inventory for measuring clinical anxiety: psychometric properties. J Consult Clin Psychol.

[B16] March JS, Parker JD, Sullivan K, Stallings P, Conners CK (1997). The Multidimensional Anxiety Scale for Children (MASC): factor structure, reliability, and validity. J Am Acad Child Adolesc Psychiatry.

[B17] Conners CK, Wells KC, Parker JD, Sitarenios G, Diamond JM, Powell JW (1997). A new self-report scale for assessment of adolescent psychopathology: factor structure, reliability, validity, and diagnostic sensitivity. J Abnorm Child Psychol.

[B18] Barkley RA, Murphy KR (1998). Attention-Deficit Hyperactivity Disorder: A Clinical Workbook.

[B19] Goodman R (1997). The Strengths and Difficulties Questionnaire: a research note. J Child Psychol Psychiatry.

[B20] Achenbach TM (1991). Manual for the Child Behavior Checklist/4–18 and 1991 Profile.

[B21] Achenbach TM (1991). Manual for the Youth Self-Report and 1991 Profile.

[B22] Sorensen MJ, Nissen JB, Mors O, Thomsen PH (2005). Age and gender differences in depressive symptomatology and comorbidity: an incident sample of psychiatrically admitted children. J Affect Disord.

[B23] Kaufman J, Volkmar MA (2007). Child Abuse and Neglect. Lewis's Child and Adolescent Psychiatry: A Comprehensive Textbook.

[B24] Thienemann M (2004). Introducing a structured interview into a clinical setting. J Am Acad Child Adolesc Psychiatry.

[B25] Hunt JI, Dyl J, Armstrong L, Litvin E, Sheeran T, Spirito A (2005). Frequency of manic symptoms and bipolar disorder in psychiatrically hospitalized adolescents using the K-SADS Mania Rating Scale. J Child Adolesc Psychopharmacol.

[B26] Soutullo CA, Chang KD, Diez-Suarez A, Figueroa-Quintana A, Escamilla-Canales I, Rapado-Castro M, Ortuno F (2005). Bipolar disorder in children and adolescents: international perspective on epidemiology and phenomenology. Bipolar Disord.

[B27] Sorensen MJ, Thomsen PH, Bilenberg N (2007). Parent and child acceptability and staff evaluation of K-SADS-PL: a pilot study. Eur Child Adolesc Psychiatry.

[B28] Carlson GA, Kashani JH, de Fatima Thomas M, Vaidya A, Daniel AE (1987). Comparison of two structured interviews on a psychiatrically hospitalized population of children. J Am Acad Child Adolesc Psychiatry.

[B29] Welner Z, Reich W, Herjanic B, Jung KG, Amado H (1987). Reliability, validity, and parent-child agreement studies of the Diagnostic Interview for Children and Adolescents (DICA). J Am Acad Child Adolesc Psychiatry.

[B30] Apter A, Orvaschel H, Laseg M, Moses T, Tyano S (1989). Psychometric properties of the K-SADS-P in an Israeli adolescent inpatient population. J Am Acad Child Adolesc Psychiatry.

[B31] Weinstein SR, Stone K, Noam GG, Grimes K, Schwab-Stone M (1989). Comparison of DISC with clinicians' DSM-III diagnoses in psychiatric inpatients. J Am Acad Child Adolesc Psychiatry.

[B32] Aronen ET, Noam GG, Weinstein SR (1993). Structured diagnostic interviews and clinicians' discharge diagnoses in hospitalized adolescents. J Am Acad Child Adolesc Psychiatry.

[B33] Vitiello B, Malone R, Buschle PR, Delaney MA, Behar D (1990). Reliability of DSM-III diagnoses of hospitalized children. Hosp Community Psychiatry.

[B34] Shanee N, Apter A, Weizman A (1997). Psychometric properties of the K-SADS-PL in an Israeli adolescent clinical population. Isr J Psychiatry Relat Sci.

[B35] Fristad MA, Cummins J, Verducci JS, Teare M, Weller EB, Weller RA (1998). Study IV: concurrent validity of the DSM-IV revised Children's Interview for Psychiatric Syndromes (ChIPS). J Child Adolesc Psychopharmacol.

[B36] Pellegrino JF, Singh NN, Carmanico SJ (1999). Concordance among three diagnostic procedures for identifying depression in children and adolescents with EBD. Journal of Emotional and Behavioral Disorders.

[B37] Kaufman J, Birmaher B, Brent D, Rao U, Flynn C, Moreci P, Williamson D, Ryan N (1997). Schedule for Affective Disorders and Schizophrenia for School-Age Children-Present and Lifetime Version (K-SADS-PL): initial reliability and validity data. J Am Acad Child Adolesc Psychiatry.

[B38] Herjanic B, Campbell W (1977). Differentiating psychiatrically disturbed children on the basis of a structured interview. J Abnorm Child Psychol.

[B39] Herjanic B, Reich W (1983). Diagnostic Interview for Children and Adolescents (DICA-C): Child version.

[B40] Herjanic B, Reich W (1983). Diagnostic Interview for Children and Adolescents (DICA-P): Parent version.

[B41] Reich W, Shayka JJ, Taibleson C (1991). Diagnostic Interview for Children and Adolescents (DICA-R-A): Adolescent version.

[B42] Reich W, Shayka JJ, Taibleson C (1991). Diagnostic Interview for Children and Adolescents (DICA-R-C): Child version.

[B43] Reich W, Shayka JJ, Taibleson C (1991). Diagnostic Interview for Children and Adolescents (DICA-R-P): Parent version.

[B44] Weller EB, Weller RA, Fristad MA, Rooney MT (1999). Children's Interview for Psychiatric Syndromes.

[B45] Costello EJ, Edelbrock CS, Costello AJ (1985). Validity of the NIMH Diagnostic Interview Schedule for Children: a comparison between psychiatric and pediatric referrals. J Abnorm Child Psychol.

[B46] Shaffer D, Fisher PW, Piacentini J, Schwab-Stone M, Wicks J (1989). Diagnostic interview schedule for children – Second revision (DISC-II).

[B47] Shaffer D, Fisher P, Dulcan MK, Davies M, Piacentini J, Schwab-Stone ME, Lahey BB, Bourdon K, Jensen PS, Bird HR (1996). The NIMH Diagnostic Interview Schedule for Children Version 2.3 (DISC-2.3): description, acceptability, prevalence rates, and performance in the MECA Study. Methods for the Epidemiology of Child and Adolescent Mental Disorders Study. J Am Acad Child Adolesc Psychiatry.

[B48] Shaffer D, Fisher P, Lucas CP, Dulcan MK, Schwab-Stone ME (2000). NIMH Diagnostic Interview Schedule for Children Version IV (NIMH DISC-IV): description, differences from previous versions, and reliability of some common diagnoses. J Am Acad Child Adolesc Psychiatry.

[B49] Bird HR, Gould MS, Staghezza B (1992). Aggregating data from multiple informants in child psychiatry epidemiological research. J Am Acad Child Adolesc Psychiatry.

[B50] Schwab-Stone ME, Shaffer D, Dulcan MK, Jensen PS, Fisher P, Bird HR, Goodman SH, Lahey BB, Lichtman JH, Canino G (1996). Criterion validity of the NIMH Diagnostic Interview Schedule for Children Version 2.3 (DISC-2.3). J Am Acad Child Adolesc Psychiatry.

[B51] Rubio-Stipec M, Canino GJ, Shrout P, Dulcan M, Freeman D, Bravo M (1994). Psychometric properties of parents and children as informants in child psychiatry epidemiology with the Spanish Diagnostic Interview Schedule for Children (DISC.2). J Abnorm Child Psychol.

[B52] Ezpeleta L, de la Osa N, Domenech JM, Navarro JB, Losilla JM, Judez J (1997). Diagnostic agreement between clinicians and the Diagnostic Interview for Children and Adolescents – DICA-R – in an outpatient sample. J Child Psychol Psychiatry.

[B53] Fristad MA, Teare M, Weller EB, Weller RA, Salmon P (1998). Study III: development and concurrent validity of the Children's Interview for Psychiatric Syndromes – parent version (P-ChIPS). J Child Adolesc Psychopharmacol.

[B54] Teare M, Fristad MA, Weller EB, Weller RA, Salmon P (1998). Study II: concurrent validity of the DSM-III-R Children's Interview for Psychiatric Syndromes (ChIPS). J Child Adolesc Psychopharmacol.

[B55] Lewczyk CM, Garland AF, Hurlburt MS, Gearity J, Hough RL (2003). Comparing DISC-IV and clinician diagnoses among youths receiving public mental health services. J Am Acad Child Adolesc Psychiatry.

[B56] Kim YS, Cheon KA, Kim BN, Chang SA, Yoo HJ, Kim JW, Cho SC, Seo DH, Bae MO, So YK (2004). The reliability and validity of Kiddie-Schedule for Affective Disorders and Schizophrenia-Present and Lifetime Version – Korean version (K-SADS-PL-K). Yonsei Med J.

[B57] Ghanizadeh A, Mohammadi MR, Yazdanshenas A (2006). Psychometric properties of the Farsi translation of the Kiddie Schedule for Affective Disorders and Schizophrenia-Present and Lifetime Version. BMC Psychiatry.

